# How level 5 leadership escalates organizational citizenship behaviour in telecom sector of Pakistan? Exploring mediatory role of organizational dissent

**DOI:** 10.1371/journal.pone.0276622

**Published:** 2022-10-21

**Authors:** Rafia Sarfraz, Kashif Rathore, Kashif Ali, Mukaram Ali Khan, Syed Sohaib Zubair

**Affiliations:** 1 Institute of Administrative Sciences, University of the Punjab, Lahore, Pakistan; 2 National School of Public Policy, Lahore, Pakistan; 3 Department of Administrative Sciences, University of the Punjab, Jhelum, Pakistan; Yunnan Technology and Business University, CHINA

## Abstract

Role of leadership in managing organizational behaviour of employees is of key essence. However, at times unconventional behaviour of employees can pose a challenge for the leaders, which in this case is organizational dissent. This study has examined the relationship between level 5 leadership and organizational citizenship behaviour (individual level-OCB-I) in presence of organizational dissent at employing a quantitative approach and a survey design. All managerial cadre employees of telecommunication sector were chosen as a population of the study. Data from 450 respondents from all four telecom companies was analysed using PLS-SEM. The findings of the study revealed that there is direct significant relationship between level 5 leadership and OCB-I of employees i.e., L5L~ OCB-I. Furthermore, the results showed an indirect relationship between level 5 leadership and OCB-I of employees through organizational dissent i.e., L5L~OD~OCB-I is significant. Telecom is a rapidly growing sector that plays a significant role in the economic growth of Pakistan. The study shows that OCB-I in telecom sector of Pakistan can be leveraged through level 5 leadership.

## 1. Introduction

Leaders play a critical role in managing organizational performance and organizational behaviour in any sector. They work to keep employee behaviour as positive in order to ensure successful contribution to the organizational excellence. However, unconventional behaviour of employees such as organizational dissent can pose a challenge for the leaders in managing workplace. Therefore, this study focuses on the relationship between two traditional concepts i.e., Level 5 Leadership (L5L) and Organizational Citizenship Behaviour at individual level (OCB-I) in the presence of Organizational Dissent (OD). OCB plays an important role in effective functioning of an organization [1]. In today’s competitive world, the organizations that have employees’ carrying out duties beyond their formal work description shown growth [2]. OCB is the discretionary and voluntary behaviour displayed by the employees. It helps in the promotion of effective working of the organization [3–5]. By discretionary, it means that the behaviour is not imposed or is not formally the part of an individual’s employment contract with organization. The individuals who display such behaviours are also referred to as ‘extra milers’ [6].

The organizational citizenship behaviour is influenced by leadership behaviours [7]. The willingness of employees to make an extra effort and go beyond formal job requirement is significantly related to the relationship between a leader and follower. The high-quality relationship between leader and follower shows increased level of citizenship behaviours [8]. Leaders are expected to deliver the vision, mission and objectives of the organization to the subordinates in a clear and concise manner, thus, increasing the creativity and innovation of employees [9]. The increase in the employees’ innovation and creativity leads to sustainable competitive advantage in today’s global market benefitting the organization [10].

Hence, important predictors of OCB are traits, skills and behaviours of leaders. Research depicts that leadership styles such as transformational, transactional, servant and leader-member exchange have a positive impact on OCB [11–15]. Moreover, the studies have reported a significant positive relationship between servant leadership and employee OCB [16–21]. When leaders act with humility which is one of the important aspects of level 5 leader, they tend to build trust among employees and employees put an extra effort in their jobs. They go beyond their required job duties and the level of creativity and innovation is also enhanced among employees [22, 23]. As discussed earlier, the idea of OCB has been widely studied in relation to traditional leadership styles, however, limited work has been conducted in the context of L5L and OCB in the telecom sector [24]. Furthermore, the said study has been conducted where OCB was analysed at organizational level and not at the individual level and that too in the presence of OD as a mediator, hence making the current research significant in terms of bridging the gap.

The next aspect of the study is to determine the influence of level 5 leadership style on the follower’s expression of dissent within organization, and *“Level 5 leaders display a powerful mixture of personal humility and indomitable will, they are incredibly ambitious and their ambition is first and foremost for the cause, organisation and its purpose and not themselves” [25].*

Employee dissent behaviour is considered to be synonymous with the employee voice behaviour, which is defined as proactively challenging the status quo and to make constructive suggestions [26]. Leaders play a critical role in the voice process. Motivation and support provided by supervisor promotes employee voice [27]. Research suggested that pro-social voice of employees can be enhanced by leadership styles such as openness of leader and leader’s positive emotions [27–29]. According to the reciprocity principle, when employees feel being valued by their leaders, they respond in return as paying back their leaders. Thus, voice behaviour of employees and similar concepts such as OCB may be considered as an important return from followers to their leaders in exchange behaviour [30]. Thus, the present study seeks to clarify the conceptual link between L5L and organizational dissent.

This study also examines the relationship between organizational dissent and OCB towards individuals. This area of research has not gained much attention of the researchers. Precisely, dissent can be referred to as an expression of contradictory views or disagreement regarding the policies and practices of an organisation [31–33]. The word ‘dissent’ is interchangeably used with whistleblowing and employee voice [34]. Therefore, relationship is established between voice behaviour of employees’ and OCB. When the employees are more involved in the work-related issues then it positively influences their attitude towards organization [35]. Precisely, this study aims to find out when employees are free to speak out their views regarding work-related issues and are valued by the leaders then employees make an extra effort and goes beyond their job requirement.

## 2. Literature review

### 2.1 Organizational citizenship behaviour

The five generally used dimensions of OCB are altruism, civic virtue, conscientiousness, sportsmanship and courtesy [36]. These dimensions represent most valid categorization of the components of OCB [37].

The five dimensions of OCB as discussed in Table 1 are further divided into two categories. The OCB’s were differentiated on the basis of the fact that who might be benefited from them [43]. OCB-O is directed towards the organization and it benefits the organization in general, whereas, OCB-I is directed at individuals and it immediately provides benefits to the individuals [43] OCB-I is defined as the workplace behaviours that are directed at certain individuals. It directly gives advantage to the individuals and also increases the organizational success indirectly [43]. OCB-O is directed towards the organization, for example, the employees agree to be a part of the organization’s work groups. They also obey organizations procedures and policies voluntarily [43]. The OCB-O demands the employees to familiarize themselves with the organization’s rules and regulations [44]. The present study is focused on the OCB-I dimension of OCB.

**Table 1:** Dimension of OCB.

Dimensions	Explanation	Reference
**Altruism**	It is the helping behaviour of the employees that goes beyond the job requirement. It includes helping co-workers in order to resolve certain difficulties that take place within the workplace. It emphasises on the behaviour that puts group concerns over individual	[36, 38]
**Civic Virtue**	It is related to the concern and indulgence that is shown by an employee during his life in the organization. It also shows employee’s commitment towards organization. These employees attend meetings regularly. They also give positive feedback and constructive suggestions at the meetings beneficial for the entire well being of the organization. The employees tend to make purposeful contributions and identifies strongly with their organization. Additionally, the employees are always concerned about the well being of the organization	[36, 38]
**Conscientiousness**	It emphasizes on dedication and responsibility. It supports the notion of adapting the behaviours that are good for the organization. It also refers to have a required level of attendance, being punctual, conserving resources, and housekeeping. It is related to the matter of internal maintenance that is beyond the minimal criteria.	[39, 40]
**Courtesy**	It refers to an employee who is courteous and avoids creating problems for his co-workers. He also helps in reducing any group conflict arising during the course of work. By this he saves managers from facing crisis management. Proactive gestures are also adapted by employees to prevent future problems. These include consulting the co-workers in the organization as well. These kinds of behaviors can be categorized as preventive measures. They are adapted to ensure the organizational effectiveness through positive communication.	[41, 42]
**Sportsmanship**	An employee who displays citizenship behaviour and can tolerate unavoidable difficulty and responsibility of work without any complain and has capability to deal grievance with co-worker. It depicts good sportsmanship. Moreover, these are the employees who can bear work settings that are not ideal. They do not get offended by the fact that other people in the organization do not support their suggestions. They also let go of their personal interests for the betterment of the organization.	[41]

### 2.2 Level 5 leadership & OCB-I

Level 5 Leaders are individuals who have a blend of extreme personal humility and intense professional will [45]. Level-5 leaders are overly ‘ambitious’ towards organizational success rather than themselves [46]. The level 5 leadership is based on two distinct dimensions i.e., personal humility (PH) and professional will (PW). The first dimension of L5L is ‘personal humility’. This concept is based on humble servant leadership. Collins described level 5 leaders as modest, humble, quiet, understated and self-effacing. Despite Collins research, his team rejected that servant leadership represent the whole concept of L5L. They believed that it may present a reasonable explanation of the first characteristic of level 5 leaders [47]. According to Collins, key characteristic of successful leadership is humility. If humility aspect is missing, the leader will impose his views on the encountered situation [48].

Level 5 leaders are also ambitious. There ambition is solely focused on the success of an organization [49]. Therefore, the second dimension of ‘professional will’ was included in the concept of L5L. Professional will is a fierce and unwavering resolve [47]. The leaders put their organizations first. They have an obsessing compulsive desire to make organization a success [49]. Furthermore, a leader with strong will would do anything to produce long term optimal outcomes for the organization, despite of any personal cost linked to it [47]. Hence, L5L has equal parts of humility and intense resolve [47].

Level 5 Leaders are highly capable individuals who can shape the behavior of employees and thus make them contribute extra to achieve organizational success [24]. L5L may facilitate workplace behaviours, support employees and play their role to increase the organizational success. Further, L5L helps to create citizenship behaviour inclined towards individuals that may include offering provision in the form of giving suggestions to other employees. Thus, L5L try to incorporate such culture within organization that help employees with relevant knowledge and skills to perform their job functions effectively. The following is hypothesized in the light of the above discussion.

**H**_**1**_: L5L positively affects OCB-I

### 2.3 Level 5 leadership & organizational dissent

Dissent can be defined as expression of contradictory views or disagreement regarding the policies and practices of an organisation [31, 32]. The dissent is expressed by members of the organizations who are more engaged in work-related issues [50]. Dissent plays a vital role in organizations. It results in improved decision-making processes and has a positive impact on organizational performance [51] and it also is seen to increase employee satisfaction [52]. Organizational dissent is described as a two-step process [53]. First, incongruence between actual and desired state is recognised by employees. This recognition of disparity by employees distances them from others that either fails to acknowledge or chooses not to see the incongruence in the organization. Dissent is only felt at this stage, but it is not expressed by the employees yet. The realization that dissent must be expressed depends on the individual level of tolerance. Once the issue at hand exceeds that level dissent is expressed [31, 53]. It must exceed the threshold level of employees which they have set regarding situations that are considered grave by them. They warrant speaking out knowing the risk associated with it [54, 55].

There are various factors that promote employee voice. One of them is the direct supervision, motivation and support for the employees [27]. Moreover, the research depicts various leadership styles such as openness of the leader and leader’s positive emotions can enhance the employee pro-social voice [28, 29]. Although there is theorising on how leadership behaviour influences the employees’ expression of contradictory opinions, but few empirical studies have been conducted to test the relationship [56].

The research conducted on authentic leadership and employee voice depicts a positive relationship [56]. Research literature reports a positive relationship between ethical leadership and employee voice [57]. Additionally, the literature on the employee voice mainly derives from the social exchange theory [29] which focuses on the fact that employees reciprocate leader’s kindness and openness to take constructive criticism with voice behaviour intended towards organisations success.

There is little research on the concept of leader’s humility which is one of the major constructs of Level 5 Leadership [58]. With the continuous development of the research literature in the management, some researchers have shed light on the concept of humble leadership in the management research field. Humble leaders have the characteristic of humility and they have a bottom-up style of leadership. Moreover, the humble leaders are modest and open to the learning of their followers and are more receptive to the new ideas proposed by employees [59]. They seek to improve the leadership effectiveness and manage staff effectively and contribute effectively towards organisations’ success [60].

There is a gap in literature and both dimensions of L5L i.e., personal humility and professional will, need to be researched in relation with dissent or similar constructs such as employee voice. The leaders’ humility is the willingness of the leader to view him accurately and the ability to appreciate the employee’s strengths and their contributions in the success of the organization. It also includes openness towards new ideas proposed by the employees and feedback [61].

Moreover, the literature suggests that employees’ constructive voice behaviour and leader humility has not been explored much [62]. The current literature indicates several desirable characteristics such as job satisfaction, work engagement and team performance are positively related to leaders’ humility [63, 64]. Leaders who are humble, acknowledge their faults and highlight the contributions of their followers promote various positive work-related outcomes such as performance and engagement. Such leaders also promote the voice behaviour of their followers. They give them support and in return the employees give constructive suggestions which enhance organizational effectiveness [58]. In literature, there exists a positive relationship between leader’s humility and employee’s constructive voice behaviour. Thus, it can be predicted that a positive relationship may exist between L5L and the acceptance of contradictory opinions regarding policies and practices of employees by the leaders such as promotion of employee’s voice behaviour or dissent.

**H**_**2**_: L5L positively affects OD

### 2.4 Organizational dissent & OCB-I

Dissent is the expression of contradictory views or disagreement with the regarding policies and practices of organisation [31, 32, 52, 65]. Some studies suggest that dissent can be considered as a constructive form of deviance [66]. The current study is considering acceptance of the constructive form of employee’s voice i.e., giving helpful suggestions and participating in the work-related issues benefitting the organization. In comparison, OCB is the discretionary and voluntary behaviour displayed by the employees. This behaviour is not a part of the formal job requirement. It helps in the promotion of effective functioning of the organization [3–5].

When employees are encouraged by the management to express their ideas related to the organizational affairs e.g., decision making, the employees get a sense of belongingness and they consider themselves as an important part of the organization [67]. The involvement of employees in affairs related to the organization positively influences their attitude towards organisation [35].

The research studies reveal that the involvement of employees and letting them voice out their opinions relating to important aspects of work-related issues such a decision making can lead to extra-role behavior by the employees. The employees took more ownership of their work and were more responsible, hence, exhibit extra role behaviour [68, 69].

The opportunity provided by the management to employees to effectively provide suggestions, opinions and criticism on the procedures and methods of the organisation implies that the management has acceptability towards the certain criticism and suggestions coming from employees and they respect the rights of the employees. The cooperative environment provided by the organization leads to increased OCB of employees [70]. The research also reveals the high-quality relationships between supervisor and the subordinates have a positive influence on the extra-role behaviour of employees [8, 71].

Moreover, dissent is also used interchangeably in literature with whistle blowing [34]. By definition, whistleblowing means to report any illegal or non-ethical action that can have a negative effect on the organisation [72]. In literature, low level and positive relationship between whistle blowing and organizational citizenship behaviour is reported [73]. There is no significant relationship between whistle blowing and OCB, but this study also reported that there was a significant relationship between two of the dimensions of these concepts, i.e., external whistle blowing and civic virtue. Although, there is less research on these concepts but as research shows certain dimension of both concepts shows significant relationships, it might create a possibility that dissent and organizational citizenship behaviour might depict a significant relationship.

It is assumed from the above discussion that the acceptance of dissent by leaders within the organization can significantly affect the employee’s citizenship behaviour by making an extra effort and helping other employees and supervisors.

***H***_***3***_: *OD positively affects OCB-I* Based on the concepts of social exchange theory, when leader puts the followers’ needs and interests first and provides assistance to the followers, help them in the work-related issues and also empower them, employees form a perception that they have been treated fairly and sincerely by the leaders. As a result, a trust is built between the leader and follower and they start viewing their relationship more in terms of a social exchange rather than an economic exchange. When the employees receive such supportive treatment by their supervisors, they engage in constructive voice behaviour and also engage in citizenship behavior [57, 74]. Following this reasoning, the current study proposes that Level 5 Leadership leads to OCB-I by the employees through the organizational dissent.

**H**_**4**_: OD mediates the relationship between L5L and OCB-I

**Fig 1:**
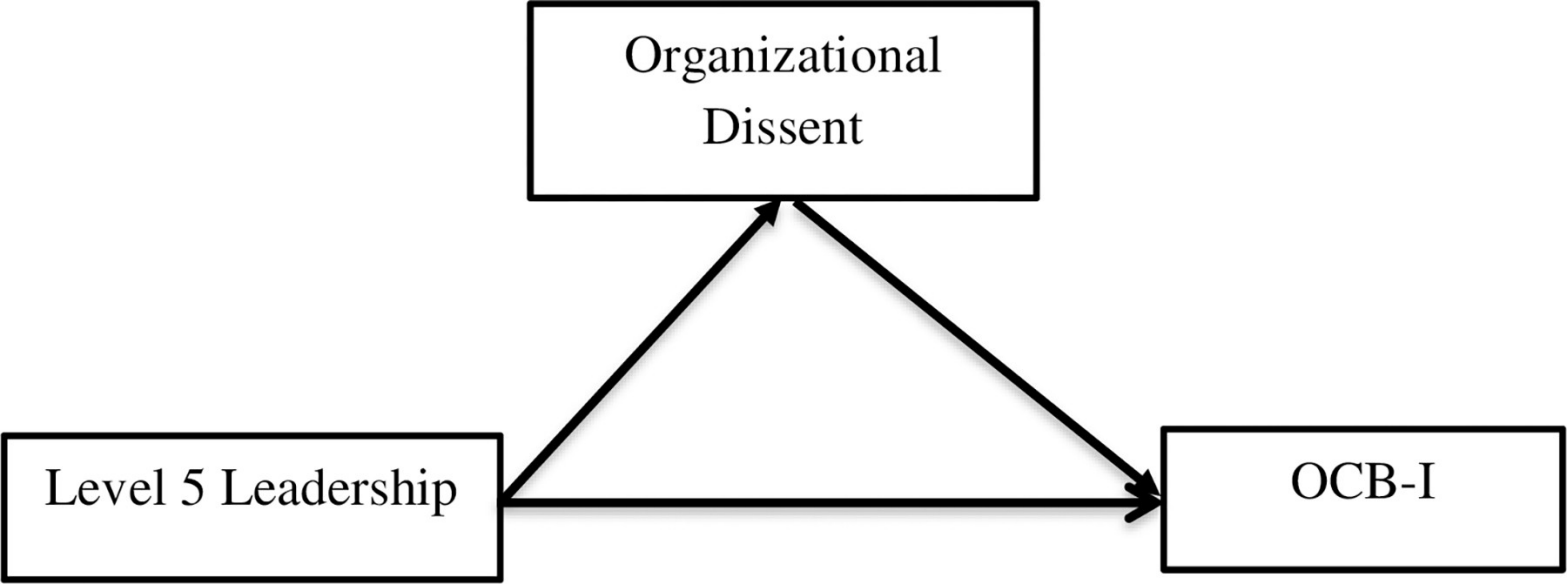
Conceptual framework.

Fig 1 shows the conceptual framework having level 5 leadership as independent variable and OCB-I as dependent variable and organizational dissent is taken as mediator, which has been influenced partially by social exchange theory.

## 3. Research methodology

Telecom sector is an important part of the service sector in Pakistan with a rapidly increasing sharing in the GDP and the Covid-19 pandemic has furthered its importance owing to the need for working from home and long-distance education. Apart from this, the telecommunication industry is chosen for the present research because of several reasons. It has emerged as an active service industry in the past three decades. The telecom sector of Pakistan plays a significant role in the growth of the economy [75]. The research is based on deductive reasoning and a survey research design is chosen. All managerial cadre employees of telecommunication sector of Pakistan are chosen as a population of the study. There are four major telecom companies that are operating all over Pakistan, i.e., Ufone, Telenor, Zong and Jazz/Warid which makes the total population of interest of the study. Since the sampling frame is available, therefore, the data is randomly collected from all of the above-mentioned companies in Lahore. The managerial level employees of telecom sector were selected from each office of the respective organization. Lahore is considered to be one of the largest cities of Pakistan, therefore, the sample has a fair chance of being representative of the population.

The sample size in the present research is calculated on the basis of item to respondent ratio, which states that the items in the scale should be multiplied with 10 in order to calculate the sample size [76]. This gives the approximate value of sample size for the present research study i.e., 32*10 = 320. For the purpose of this study, a total of 550 questionnaires were distributed in the four above mentioned telecom companies. Out of 550 questionnaires, 485 questionnaires were returned by the respondents which give us a response rate of 88%. 35 questionnaires were discarded because they were incomplete and filled incorrectly. Therefore, the sample size of 450 is taken for the purpose of this research which is more than the approximate value calculated by the item to respondent ratio. For data collection, already developed measures are used. For the measurement of Level 5 Leadership, a 10-item scale “1 being least and 10 being highest” developed by Reid III, Bud West, Winston & Wood has been used [77]. Organisational dissent is measured using a five-point Likert scale [strongly disagree (1), disagree (2), neutral (3), agree (4), strongly agree (5)] developed by Kassing and has 15 items [34]. Organisational dissent is measured on three different dimensions which are articulated or upward dissent (AD), latent or lateral dissent (LD) and displaced outward dissent (DD). OCB-I, is measured using a 5-point Likert scale developed by Williams & Anderson [43]. OCB-I is measured by seven items. It is imperative to mention that verbal informed consent was taken from the respondents and all details regarding the purpose of data collection and the research work were shared in a cover letter attached with the instrument. The study is a non-funded independent in nature and does not in any way affect any participant or any institution. Moreover, this research also did not require any observation or any experimental intervention towards the participant. Therefore, owing to these reasons, in the regulatory framework or scope of this country, the authors/researchers do not need any approval or permission from any authority apart from getting informed consent from the individuals who provide data in the form of questionnaires., hence no prior board or committee’s approval was required in the said case.

## 4. Data analysis

The data shows that 57.80% participants are male and 42.20% are female respondents in the sample. The age of the respondents is categorized into eight (08) categories which shows that data is collected from all age groups. Level of employment is categorized into three groups i.e., senior, middle and junior management as shown in Table 2 along other descriptives.

**Table 2:** Descriptive analysis.

		%	Mean	Std. deviation	Skewness	Kurtosis
**Age**	less than 25	14.90				
	26–30	26.20				
	31–35	28.40				
	36–40	17.60				
	41–45	6.70				
	46–50	2.70				
	51–55	2.40				
	More than 55	1.10				
**Gender**	Male	57.80				
	Female	42.20				
**Education**	Undergraduate (14 years)	29.10				
	Masters (16 years)	54.20				
	Post graduate (18 years)	14.00				
	More than 18 years	2.70				
**Level of Employment**	Senior Management	15.80				
	Middle Management	50.40				
	Junior Management	33.80				
**PH**			5.69	2.12	-0.17	-0.73
**PW**			5.58	2.03	-0.16	-0.73
**L5L**			5.60	1.90	-0.18	-0.68
**AD**			3.17	1.04	-0.22	-0.52
**LD**			3.18	1.07	-0.31	-0.55
**DD**			3.18	0.96	-0.38	-0.28
**OD**			3.17	1.00	-0.31	-0.53
**OCB**			3.08	0.74	-0.15	-0.26

### 4.1 Partial Least Square Structural Equation Modelling (PLS-SEM)

In order to develop and test the model or test the hypothesis, PLS-SEM has been used. As compared to CB-SEM, PLS-SEM provides less rigid sample size and model fitness restrictions. “PLS-SEM works efficiently with relatively small sample sizes and complex models and makes practically no assumptions about the underlying data [distributions]” [78].

Data in PLS-SEM method is analysed in two steps, firstly, path model or measurement model is analysed and after meeting certain criteria of measurement model the structural model parameters are assessed.

To deal with the potential common method bias (CMB) issue, researchers have used a full collinearity assessment approach [79]. If all the constructs VIF values are less than 3.3, then it can be concluded that the data is free from CMB, as is the case in this study.

#### 4.1.1 Estimation of measurement model

The value of the composite reliability and Cronbach Alpha should be 0.7 or higher for an adequate model for confirmatory research purpose [80]. Table 3 shows that all the items are reporting higher values according to the required benchmark that is considered to be a good fit for the confirmatory research purposes as referred above, satisfying the internal consistency reliability criteria.

**Table 3:** Cronbach Alpha, composite reliability and AVE.

Constructs	Cronbach’s Alpha	Composite Reliability	Average Variance Extracted (AVE)
**AD**	0.792	0.812	0.761
**DD**	0.756	0.811	0.746
**LD**	0.760	0.816	0.757
**OD**	0.817	0.852	0.781
**PH**	0.800	0.820	0.848
**PW**	0.776	0.821	0.762
**L5L**	0.808	0.828	0.784
**OCB**	0.775	0.835	0.692

**Fig 2:**
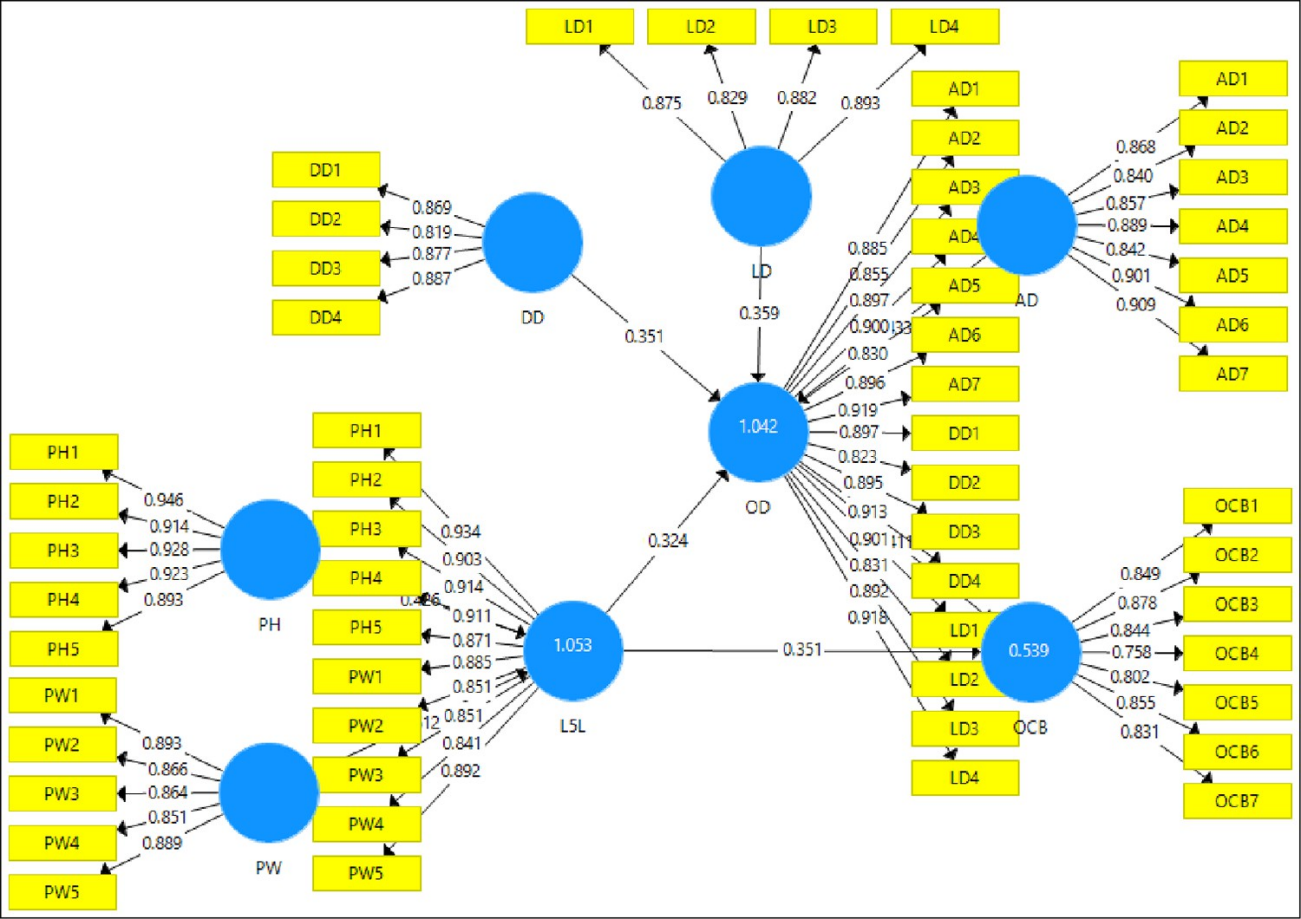
Factor loadings.

The convergent validity is supported when the values of outer loadings are above 0.70 and the value of AVE is 0.50 or higher [81]. All outer loadings of the variables are more than 0.70 as shown in Fig 2. Moreover, the value of 0.50 of AVE indicates that the construct explains more than half of the variance of its indicators. Table 3 shows that all the indicators have AVE greater than 0.50 [81] which is the required level, it means the construct explains more than half of the variance of its indicators and it also satisfies the required benchmark of convergent validity.

From Table 4, it can be assessed that all the constructs of the study have values of square root of AVE that are higher than the latent variable correlations and fulfills the criteria of discriminant validity. Moreover, cross loadings of the indicators of the constructs of the study to their latent variable. All the values are higher than any another constructs and it is in accordance with the given criteria of discriminant validity.

**Table 4:** Discriminant validity—Fornell-larcker criterion.

	AD	DD	L5L	LD	OCB	OD	PH	PW
**AD**	(0.873)							
**DD**	0.341	(0.864)						
**L5L**	0.371	0.351	(0.886)					
**LD**	0.443	0.475	0.255	(0.870)				
**OCB**	0.345	0.245	0.539	0.238	(0.832)			
**OD**	0.626	0.594	0.446	0.643	0.428	(0.884)		
**PH**	0.256	0.336	0.610	0.240	0.422	0.331	(0.921)	
**PW**	0.362	0.341	0.578	0.345	0.424	0.336	0.454	(0.873)

Degree of redundancy is measured with the help of Variance Inflation Factors (VIF) and all values are less than 5 which fulfil the criteria [82].

Fig 2 shows the factor loadings of the model and it shows that all the factor loadings are more than the defined threshold value as discussed above.

### 4.2 Assessment of structural model

Before examining the structural model, the level of collinearity needs to be determined in the structural model under study [78]. VIF values (AD = 2.88, DD = 1.54, LD = 2.02, PH = 1.68, PW = 1.07) indicates that there are no problems of multi-collinearity in the inner model, as the values of VIF (variance inflation factor) are below the threshold of 5 [82].

#### 4.2.1 Direct effects of the model

The Table 5 shows direct effects of the model. It reveals that L5L has positive significant influence on OCB-I i.e., b = 0.351, t = 6.774, p < 0.001. L5L has positive significant influence on organizational dissent i.e., b = 0.324, t = 4.991, p < 0.001. Organizational dissent has positive significant influence on OCB-I i.e., b = 0.433, t = 5.040, p < 0.001.

**Table 5:** Results of direct effects.

	Path Coefficients	Standard Deviation (STDEV)	t statistics (|O/STDEV|)	p values	Decision
**L5L -> OCB**	0.351	0.081	6.774	p < 0.001	Supported
**L5L -> OD**	0.324	0.036	4.991	p < 0.001	Supported
**OD -> OCB**	0.433	0.074	5.040	p < 0.001	Supported

**Fig 3:**
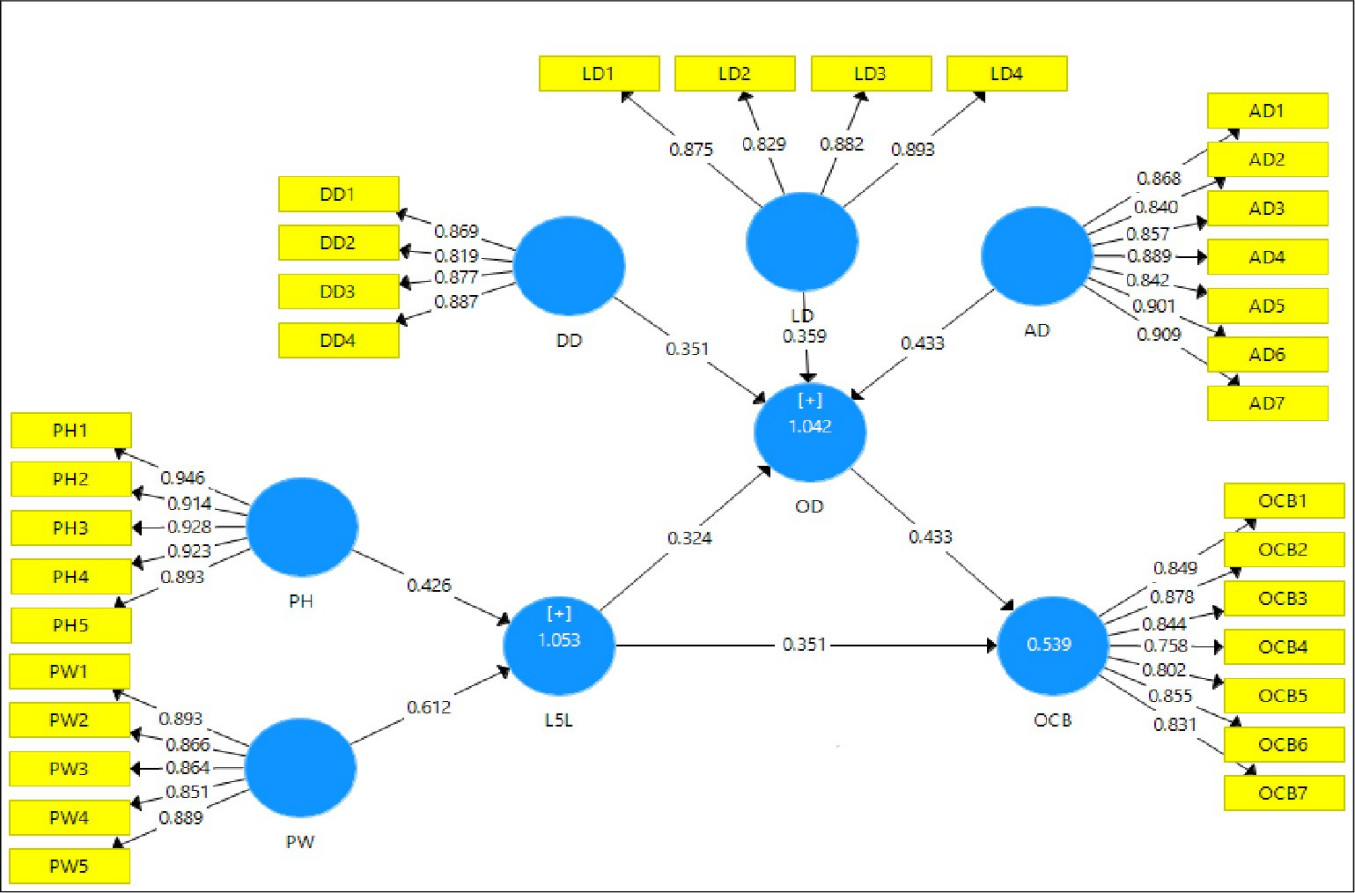
Path coefficients of the model.

Fig 3 shows the path coefficients of the model which are added in Table 5 and discussed above. It shows that all the path coefficients are significant.

**Fig 4:**
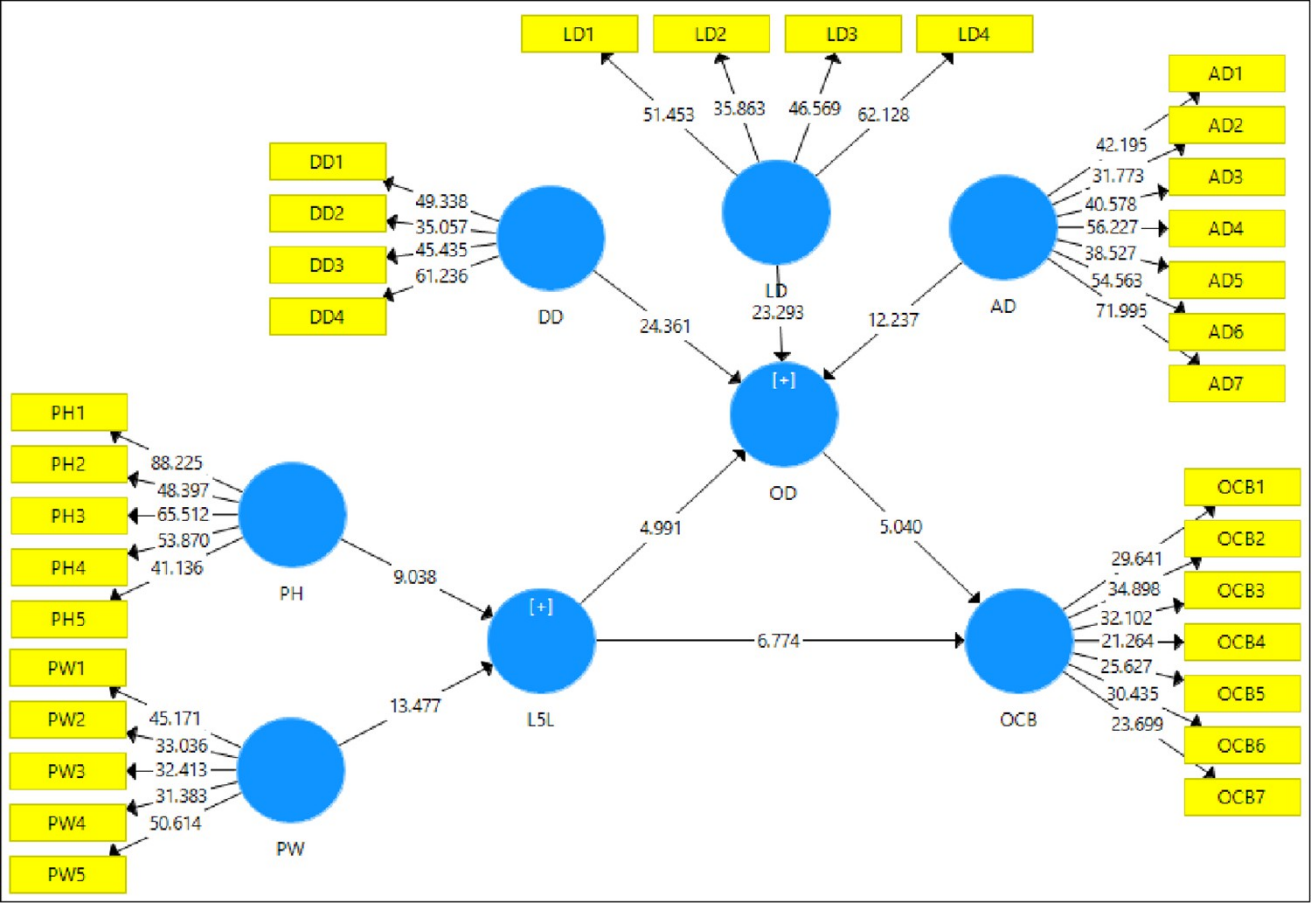
T-statistic of the model.

Fig 4 shows the t statistic of the model which are added in Table 5 and discussed above. All the t values are higher than the threshold values of 1.96 showing significant path coefficients.

#### 4.2.2 Indirect effect and total effect calculations

Indirecteffect=a*b

Indirecteffect=0.324*.433=0.140

Totaleffect=Indirecteffect+Directeffect

Totaleffect=0.140+0.351=0.491

$$VAF=\frac{Indirect\;effect}{Total\;effect}*100$$

$$VAF=\frac{0.140}{0.491}*100=28.5\%$$

#### 4.2.3 Indirect path L5L -> OD -> OCB, t value


$$t\;value=\frac{Indirect\;effect}{standard\;deviation}$$

$$t\;value=\frac{0.140}{0.0547}=2.559$$

Thus, the above calculation reveals that 28.5% of the effect of L5L on OCB-I is explained through organisational dissent. VAF demonstrates partial mediation when threshold level is exceeded by 0.2 and it demonstrates full mediation when threshold level of 0.8 is exceeded [82]. Since, the value of VAF is between 20% and 80% organisational dissent partially mediates the relationship between L5L and OCB-I. Thus, these findings lead us to support Hypothesis 4.

## 5. Discussion

Since the early 2000s, Pakistan’s telecom sector has experienced fast growth and has been essential to the country’s economic expansion. A nation’s economy, culture, and social development are said to be determined by its level of telecommunication sector’s development. Pakistan, a middle-income nation, has seen political strife but has nonetheless managed to build its telecommunications industry quickly, maintaining its position as Asia’s fastest-growing telecom market. Cellular mobile telecommunication services were established in the early 1990s, and today Pakistan’s telecom sector is one of the biggest providers of low-cost calling rates in the region [83]. In Pakistan’s mobile telecommunications sector, the five major carriers are now engaged in a fierce battle. These carriers are developing strategies focused on providing the most affordable calling rates, comprehensive coverage, connection, and cutting-edge value-added services. More than 25,000 experts work in Pakistan’s mobile telecommunications sector. Due to the ongoing importance, the results of this study are useful in number of ways as discussed below.

The current study has examined the helping behaviour of employees’ (OCB-I) influence by level 5 leaders’ through organizational dissent in the telecommunication sector of Pakistan. The study makes a significant contribution to existing literature on L5L and its relationship with organizational citizenship behaviour towards employees as there is only one study conducted previously [19]. The L5Ls’ are very ambitious [46], supportive, motivational and empower their employees. Such leaders provide a supportive environment to their followers and give them credit of the organizational success. The Level 5 Leaders are humble leaders and are ambitious towards achieving organisational goals. They are modest and at the same time are fierce when it comes to achieving organisational objectives. Rather they lead by example and prepare successors which will prove successful for the organisation in future [84]. The present study shows that when the leader provides such a supportive environment to employees’ and make them feel valued in the organization, the employees also show a positive response by making an extra effort. As a result, their helping behaviour enhances and they tend to help their supervisors and colleagues, share their burden and help them understand certain aspects of work by making an extra effort apart from their formal job requirement. The analysis of L5L with the OCB-I reveals *L5L positively affects OCB-I*, accepting first hypothesis (H_1_) of the study. Literature on Level 5 leadership also confirms the current findings. The literature corroborates with our findings as it stated that the level 5 leaders are capable of moulding the behaviours of their followers and thus can make the employees to contribute extra effort in order to achieve organisational effectiveness [24].

Moving on, this study also examined the relationship between organisational dissent and L5L. Dissent and voice are alternatively used in the literature [34]. Voice is a broader concept as compared to dissent. Voice is distinguished by dissent relating to the audience it addresses and the nature of arguments. Voice is related to challenging the supervisor or when there is internal communication. Arguments can be related to any sort of agreement, disagreement or providing support or engaging employees related to work-place issues increasing organisation’s effectiveness. In contrast, in organizational dissent communication takes place in all directions and the employees’ express disagreements only that are related to work-place policies or practices to achieve organisational efficiency [34, 85, 86]. Hence, both the concepts promote organisational efficiency. The employee voice is promoted by various factors [87]. One of the factors to promote such behaviour of employees’ is the motivation and support provided by the supervisor [27]. Moreover, L5Ls dimension humility is taken up instead of L5L, as no direct article is there in literature which provides evidence on relating dissent and L5L. The concept of L5L is based on two dimensions; personal humility and professional [47]. The leaders’ having the characteristic of humility are open to learning and receptive to new ideas. They are humble and modest leaders [59].

The research also validated various leadership styles such as leaders’ openness and leaders’ positive emotions enhances employee pro-social voice [27–29]. The present study has made use of the concept of employee voice and leaders’ humility in order to build up the theoretical argument which can justify the current study.

The current literature indicates various desirable characteristics such as job satisfaction, work engagement and team performance to have a positive relationship with the leaders’ humility [63, 64]. Leaders who are humble and acknowledge their faults and highlight the contributions of their followers promote various positive work-related outcomes such as performance and engagement. Such leaders also promote the voice behaviour of their followers. They give them support and in return the employees give constructive suggestions which may enhance organisational effectiveness [58]. The literature showed a positive relationship between the constructive voice behaviour of employees’ and leaders’ humility [62]. The results of the study indicate that when the leaders are supportive towards employees’ learning and have an acceptance to new ideas and suggestions the employees’ voice out their opinions more frequently. Thus, the result confirms that *L5L positively affects OD*, which accepts the second hypothesis (H_2_) of the study.

Moreover, the research study has examined the relationship between organizational dissent and OCB-I of employees. The previous theorising on OCB-I and organisational dissent reveals that both are considered to be extra-role behaviours, not a part of formal job requirement. There is a distinction in both the concepts in literature. The OCB-I is concerned with the helping behaviour of employees [43]. In contrast, the employees expressing disagreement towards certain policies and practices related to organisation is known as dissent [31, 32]. Dissent and voice are interchangeably used in literature as explained in earlier sections of the study. The concept of voice is incorporated to build a relationship between OCB-I and organisational dissent. Voice behaviour of employees was said to be one of the dimensions of classification of OCB known as challenge-oriented citizenship behaviour (COCB) [88] and is also related to organisational citizenship behaviour towards organisation (OCB-O) [89]. Hence, there is a clear distinction between OCB-I and organisational dissent in literature. The present study strives to find out the relationship between the extra role behaviour related to helping behaviour of employees’ and the expression of contradictory opinions relating to organisations policies and practices by employees. The results of the study reveal a significant relationship between OCB-I and organisational dissent. When the employees were given an opportunity to voice out their opinions and suggestions, made part of decision-making process they felt more involved [67]. The previous research findings corroborate with the present study and hence, endorse the third hypothesis (H_3_).

The structural model examined through PLS-SEM shows that L5L is significantly correlated with OCB-I of the employees mediated by organizational dissent that supports H_4_. It can be argued that those leaders who act with modesty and humility, show firm determination in achieving organisational objectives, welcome employees to express contradictory views relating to work-related issues. As a result of this leadership behaviour, the employees’ feel valued in the organisation as they are considered a part of organisations well-being, a feeling of belongingness is generated, the employees’ in return makes an extra effort to help their fellow workers and supervisors in work-related tasks. This overall increases the organisational efficiency. Previous literature has explained similar relationships with the help of social exchange theory [57, 74]. Thus, the findings of the study are supported by the previous literature and demonstrate a significant relationship between L5L, organisational dissent and organisational citizenship behaviour towards employees. Summary of the results has been given below in Table 6.

**Table 6:** Results.

Sr.#	Hypothesis	Supported/ not supported
1	*L5L positively affects OCB-I*	Supported
2	*L5L positively affects OD*	Supported
3	*OD positively affects OCB-I*	Supported
4	*OD mediates the relationship between L5L and OCB-I*	Supported

### 5.1 Conclusion

The findings of the research study indicate that organizational citizenship behaviour of employees’ can be enhanced by level 5 leaders. The level 5 leaders act with humility and modesty towards their employees. They also depict firm determination in achieving organisational objects. They use participative mode of leadership, promote teamwork, assist and motivate their employees’ and are open to criticism and suggestions for the improvement of organisational effectiveness. Such constructive and supportive behaviour by the leaders promotes citizenship behaviour among employees. The employees make an extra effort and go beyond their job role and requirement and share the burden of their supervisor and colleagues. The level 5 leaders are directed to turn good organisations into great ones and this require moulding the behaviours of employees in achieving organisational efficiency as employees are an important part of an organisation. As today’s business environment is competitive and challenging and with various social and psychological pressures on employees owing to the pandemic, the leadership style needs to be democratic and employee oriented. The present study favours such leadership style and also states its benefits for organisational effectiveness and success.

### 5.2 Managerial and theoretical implications

As the fundamental concept under review i.e. Level 5 Leadership is a relatively new concept, this research definitely adds to the theoretical and conceptual understanding of this idea by contributing to the fundamental literature and social exchange theory. Moreover, as for the managerial aspect, it suggests managers to explore a new technique or genre of leadership that can positively contribute in the much-needed organization citizenship behaviour, however in the presence of dissenting behaviors or attitudes, the said relationship is reversed, hence generating negative vibes in the organizational environment. Therefore, the study advises managers to be vigilant in identifying and engaging dissenting ideas or behaviours in order to manage them in a better way.

### 5.3 Limitations & future research directions

The data collected in this study is primarily from service industry, i.e., telecom sector which restricts its application to other sectors and different industries. Moreover, the study involved the data collection from only one city of Pakistan, Lahore. Therefore, it limits the generalizability all over Pakistan and across different sectors.

The current research is based on cross-sectional design and a quantitative approach. Similar research can be conducted by taking the same variables ‘L5L, organisational dissent and OCB-I’ with qualitative approach and longitudinal design. A study on L5L at the levels of CEOs is recommended to further explore the concept of L5L following Jim Collins methodology [90]. The variables used in this study have contributed in the literature of L5L and OCB; still several other variables such as OCB-O can be further explored. The future research may also include industry wise analysis. By this, differences across different industries can be captured and analysed.

## Supporting information

S1 File (SAV)

## References

[1] Hemaloshinee V. A. (2021). Examining the Relationship between Organizational Commitment and Non-Supervisory Organizational Citizenship Behaviour. *INTI JOURNAL*, 2

[2] Ali U., & Waqar S. (2013). Teachers’ organizational citizenship behavior working under different leadership styles. *Pakistan Journal of Psychological Research*, 28(2), 297–316.

[3] Appelbaum S., Bartolomucci N., Beaumier E., Boulanger J., Corrigan R., Dore I., et al. (2004). Organizational citizenship behavior: a case study of culture, leadership and trust. *Management decision*, 42(1), 13–40.

[4] Robbins S. P., & Judge T. (2012). *Essentials of organizational behavior*. United Kingdom: Pearson Education Limited.

[5] Cichorzewska, M., & Rakowska, A. (2017). *Organizational citizenship behavior of Polish and Ukrainian civil servants–a comparative study*. Paper presented at the Management Challenges in a Network Economy: Proceedings of the MakeLearn and TIIM International Conference.

[6] Cross R., Rebele R., & Grant A. (2016). Collaborative overload. *Harvard Business Review*, 94(1).

[7] Bantha T., & Sahni S. P. (2021). The relation of servant leadership with followers’ organizational citizenship behaviour (OCB): mediating role of generalized self-efficacy (GSE) and organization–based self-esteem (OBSE). *Industrial and Commercial Training*, 53(4), 331–342.

[8] Isiaka S., Adeoti J. O., & Aliyu M. (2019). Leader-Member Exchange and Organisational Citizenship Behaviour in Tuyil Pharmaceutical Industry, Nigeria. *Nigeria* *(*January 17, 2019).

[9] Afsar B., & Umrani W. A. (2019). Transformational leadership and innovative work behavior: The role of motivation to learn, task complexity and innovation climate. *European Journal of Innovation Management*, 23(3), 402–428.

[10] Al-Khasawneh A. L., & Moh’d Futa S. (2013). The impact of leadership styles used by the academic staff in the Jordanian public universities on modifying students’ behavior: A field study in the northern region of Jordan. *International Journal of Business and Management*, 8(1), 1–10.

[11] Purwanto A. (2022). The Role of Transformational Leadership and Organizational Citizenship Behavior on SMEs Employee Performance. *Journal of Industrial Engineering & Management Research*. 3(5), 39–45.

[12] Novianti K. R. (2021). Does Organizational Commitment Matter? Linking Transformational Leadership with Organizational Citizenship Behavior (OCB). *Jurnal Aplikasi Manajemen*, 19(2), 335–345.

[13] Gurmani J. K., Khan N. U., Khalique M., Yasir M., Obaid A., & Sabri N. A. A. (2021). Do environmental transformational leadership predicts organizational citizenship behavior towards environment in hospitality industry: using structural equation modelling approach. *Sustainability*, 13(10), 5594.

[14] Engelbrecht A. S., & Schlechter A. F. (2006). The relationship between transformational leadership, meaning and organisational citizenship behaviour. *Management Dynamics*: *Journal of the Southern African Institute for Management Scientists*, 15(4), 2–16.

[15] Emami M., Alizadeh Z., Nazari K., & Darvishi S. (2012). Antecedents and consequences of organisational citizenship behaviour (OCB). *Interdisciplinary Journal of Contemporary Research in Business*, 3(9), 494–505.

[16] Liden R. C., Wayne S. J., Zhao H., & Henderson D. (2008). Servant leadership: Development of a multidimensional measure and multi-level assessment. *The leadership quarterly*, 19(2), 161–177.

[17] Vondey M. (2010). The relationships among servant leadership, organizational citizenship behavior, person-organization fit, and organizational identification. *International Journal of Leadership Studies*, 6(1), 3–27.

[18] Walumbwa F. O., Hartnell C. A., & Oke A. (2010). Servant leadership, procedural justice climate, service climate, employee attitudes, and organizational citizenship behavior: a cross-level investigation. *Journal of applied psychology*, 95(3), 517. doi: 10.1037/a0018867 20476830

[19] Bambale A. J. A., Shamsudin F. M., Chandrakantan A., & Subramaniam L. (2011). Stimulating organizational citizenship behavior (OCB) research for theory development: Exploration of leadership paradigms. *International journal of academic research in business and social sciences*, 1, 48.

[20] Newman A., Schwarz G., Cooper B., & Sendjaya S. (2017). How servant leadership influences organizational citizenship behavior: The roles of LMX, empowerment, and proactive personality. *Journal of Business Ethics*, 145(1), 49–62.

[21] Qiu S., & Dooley L. (2022). How servant leadership affects organizational citizenship behavior: the mediating roles of perceived procedural justice and trust. *Leadership & Organization Development Journal*. 43(3), 350–369.

[22] Pfeffer J., & Jeffrey P. (1998). *The human equation*: *Building profits by putting people first*: Harvard Business Press.

[23] Block P. (2013). *Stewardship*: *Choosing service over self-interest*: Berrett-Koehler Publishers.

[24] Shehada M., & Dawod W. Y. (2015). The Relationship between Managers’ Level-Five Leadership Style and their Employees’ Organizational Citizenship Behavior in the Telecommunication Companies in Jordan. *European Journal of Business and Management*, 7(9),63–85.

[25] Collins J. (2007). Level 5 leadership. *The Jossey-Bass reader on educational leadership*, 2, 27–50.

[26] Van Dyne L., & LePine J. A. (1998). Helping and voice extra-role behaviors: Evidence of construct and predictive validity. *Academy of Management journal*, 41(1), 108–119.

[27] Detert J. R., & Burris E. R. (2007). Leadership behavior and employee voice: Is the door really open? *Academy of Management journal*, 50(4), 869–884.

[28] Liu W., Zhu R., & Yang Y. (2010). I warn you because I like you: Voice behavior, employee identifications, and transformational leadership. *The leadership quarterly*, 21(1), 189–202.

[29] Liu W., Tangirala S., Lam W., Chen Z., Jia R. T., & Huang X. (2015). How and when peers’ positive mood influences employees’ voice. *Journal of applied psychology*, 100(3), 976. doi: 10.1037/a0038066 25365730

[30] Yan A., & Xiao Y. (2016). Servant leadership and employee voice behavior: a cross-level investigation in China. *SpringerPlus*, 5(1), 1595. doi: 10.1186/s40064-016-3264-4 27652168 PMC5026985

[31] Redding W. C. (1985). Rocking boats, blowing whistles, and teaching speech communication.

[32] Kassing J. W., & DiCioccio R. L. (2004). Testing a workplace experience explanation of displaced dissent. *Communication Reports*, 17(2), 113–120.

[33] Özgenel M., & Çetin M. (2021). Effects of organizational cynicism occupational commitment and organizational dissent on knowledge inertia. *Kalem Eğitim ve İnsan Bilimleri Dergisi*, 11(2), 365–389.

[34] Kassing J. W. (1998). Development and validation of the organizational dissent scale. *Management Communication Quarterly*, 12(2), 183–229.

[35] Hendry C. (2012). *Human resource management*. Routledge.

[36] Organ D. W. (1988). *Organizational citizenship behavior*: *The good soldier syndrome*: Lexington Books/DC Heath and Com.

[37] Kashif M., Khan Y., & Rafi M. (2011). An Exploration of the Determinants of OCB in the Telecommunication Sector of Pakistan. *Asian Journal of Business Management*, 3(2), 91–97.

[38] Redman T., & Snape E. (2005). I to wed: The role of consciousness transformation in compassion and altruism. *Journal of Management Studies*, 42(2), 2200–2380.

[39] King E. B., George J. M., & Hebl M. R. (2005). Linking personality to helping behaviors at work: An interactional perspective. *Journal of Personality*, 73(3), 585–608. doi: 10.1111/j.1467-6494.2005.00322.x 15854007

[40] Bateman T. S., & Organ D. W. (1983). Job satisfaction and the good soldier: The relationship between affect and employee “citizenship”. *Academy of Management journal*, 26(4), 587–595.

[41] Podsakoff P. M., & MacKenzie S. B. (1997). Impact of organizational citizenship behavior on organizational performance: A review and suggestion for future research. *Human performance*, 10(2), 133–151.

[42] Geçkil T., & Tikici M. (2015). Organisational Democracy Scale. *Amme Administration Journal*, 48(4).

[43] Williams L. J., & Anderson S. E. (1991). Job satisfaction and organizational commitment as predictors of organizational citizenship and in-role behaviors. *Journal of management*, 17(3), 601–617.

[44] Podsakoff P. M., MacKenzie S. B., Paine J. B., & Bachrach D. G. (2000). Organizational citizenship behaviors: A critical review of the theoretical and empirical literature and suggestions for future research. *Journal of management*, 26(3), 513–563.

[45] Collins J. (2001). *Why some companies make the leap… and others don’t. Good to Great*. New York: Harperbusiness.

[46] Itoya J., & Igbokwe I. C. (2020). Level-5 and Charismatic Leadership Styles and Employees’performance. *European Journal of Social Sciences Studies*, 5(4), 49–61.

[47] Collins J. (2001). *Level 5 Leadership*: *The Triumph of Humility and Fierce Resolve*. 11189464

[48] Serfontein K., & Hough J. (2011). Nature of the relationship between strategic leadership, operational strategy and organisational performance. *South African Journal of Economic and Management Sciences*, 14(4), 393–406.

[49] Collins J. (2009). Good to Great-(Why Some Companies Make the Leap and others Don’t): SAGE Publications Sage India: New Delhi, India.

[50] Kassing J. W., Piemonte N. M., Goman C. C., & Mitchell C. A. (2012). Dissent expression as an indicator of work engagement and intention to leave. *The Journal of Business Communication* *(*1973*)*, 49(3), 237–253.

[51] Ng T. W., & Feldman D. C. (2012). Employee voice behavior: A meta‐analytic test of the conservation of resources framework. *Journal of Organizational behavior*, 33(2), 216–234.

[52] Alniaçik E., & Kelebek E. F. E. (2021). The Effect of Organizational Dissent on Affective Commitment and Job Satisfaction. *Journal of Economics and Research*, 2(1), 1–12.

[53] Kassing J. W. (1997). Articulating, antagonizing, and displacing: A model of employee dissent. *Communication Studies*, 48(4), 311–332.

[54] Kassing J. W., & Armstrong T. A. (2002). Someone’s going to hear about this: Examining the association between dissent-triggering events and employees’ dissent expression. *Management Communication Quarterly*, 16(1), 39–65.

[55] Kassing J. W. (2009). “In Case You Didn’t Hear Me the First Time” An Examination of Repetitious Upward Dissent. *Management Communication Quarterly*, 22(3), 416–436.

[56] Hsiung H.-H. (2012). Authentic leadership and employee voice behavior: A multi-level psychological process. *Journal of Business Ethics*, 107(3), 349–361.

[57] Walumbwa F. O., & Schaubroeck J. (2009). Leader personality traits and employee voice behavior: mediating roles of ethical leadership and work group psychological safety. *Journal of applied psychology*, 94(5), 1275. doi: 10.1037/a0015848 19702370

[58] Liu C. (2016). Does humble leadership behavior promote employees’ voice behavior? A dual mediating model.

[59] Owens B. P., & Hekman D. R. (2016). How does leader humility influence team performance? Exploring the mechanisms of contagion and collective promotion focus. *Academy of Management journal*, 59(3), 1088–1111.

[60] Nielsen R., Marrone J. A., & Slay H. S. (2010). A new look at humility: Exploring the humility concept and its role in socialized charismatic leadership. *Journal of Leadership & Organizational Studies*, 17(1), 33–43.

[61] Owens B. P., Johnson M. D., & Mitchell T. R. (2013). Expressed humility in organizations: Implications for performance, teams, and leadership. *Organization Science*, 24(5), 1517–1538.

[62] Li J., Liang Q., Zhang Z., & Wang X. (2018). Leader humility and constructive voice behavior in China: a dual process model. *International Journal of Manpower*, 39(6), 840–854.

[63] Ou A. Y., Tsui A. S., Kinicki A. J., Waldman D. A., Xiao Z., & Song L. J. (2014). Humble chief executive officers’ connections to top management team integration and middle managers’ responses. *Administrative Science Quarterly*, 59(1), 34–72.

[64] Rego A., Owens B., Yam K. C. S., Bluhm D., Cunha M. P., Silard A., et al. (2017). Leader humility and team performance: Exploring the mechanisms of team psychological capital and task allocation effectiveness. *Journal of management*.

[65] Kassing J. W. (2011). Stressing out about dissent: Examining the relationship between coping strategies and dissent expression. *Communication Research Reports*, 28(3), 225–234.

[66] Warren D. E. (2003). Constructive and destructive deviance tn organizations. *Academy of management Review*, 28(4), 622–632.

[67] Tutar H., & Sadykova G. (2014). A study on the relationship between organizational democracy and organizational opposition. *Journal of Business Administration*, 2(1), 1–16.

[68] Harrison J. S., & Freeman R. E. (2004). Is organizational democracy worth the effort? *The Academy of Management Executive (1993–2005)*, 49–53.

[69] Porter L. W., Lawler E. E., & Hackman J. (1996). Ways groups influence individual work effectiveness. *Motivation and leadership at work*, 346–354.

[70] Tokay Ö., & Eyupoglu S. Z. (2018). Employee perceptions of organisational democracy and its influence on organisational citizenship behaviour. *South African Journal of Business Management*, 49(1), 9.

[71] Ilies R., Nahrgang J. D., & Morgeson F. P. (2007). Leader-member exchange and citizenship behaviors: A meta-analysis. *Journal of applied psychology*, 92(1), 269. doi: 10.1037/0021-9010.92.1.269 17227168

[72] Miceli M. P., Near J. P., & Dworkin T. M. (2008). *Whistle-blowing in organizations*: Psychology Press.

[73] Ozsoy N., & Beduk A. (2015). The relationship between whistleblowing and organizational citizenship behaviour. *International journal of academic research in business and social sciences*, 5(4), 146–156.

[74] Walumbwa F. O., Morrison E. W., & Christensen A. L. (2012). Ethical leadership and group in-role performance: The mediating roles of group conscientiousness and group voice. *The leadership quarterly*, 23(5), 953–964.

[75] Javed A. (2020). The Scope of Information and Communication Technology Enabled Services in Promoting Pakistan Economy. *Asian Journal of Economics*, *Finance and Management*, 2(4), 1–9.

[76] Hair J. F., Anderson R. E., Babin B. J., & Black W. C. (2010). Multivariate data analysis: A global perspective (Vol. 7): Upper Saddle River, NJ: Pearson.

[77] Reid W. A. III, Bud West G., Winston B. E., & Wood J. A. (2014). An instrument to measure level 5 leadership. *Journal of Leadership Studies*, 8(1), 17–32.

[78] Hair J. F. Jr, Sarstedt M., Hopkins L., & Kuppelwieser V. G. (2014). Partial least squares structural equation modeling (PLS-SEM). *European business review*.

[79] Kock N. (2015). Common method bias in PLS-SEM: A full collinearity assessment approach. *International Journal of e-Collaboration*, 11(4), 1–10.

[80] Henseler J., Ringle C. M., & Sinkovics R. R. (2009). The use of partial least squares path modelling in international marketing *New challenges to international marketing* (pp. 277–319): Emerald Group Publishing Limited.

[81] Bagozzi R. P., & Yi Y. (1988). On the evaluation of structural equation models. *Journal of the academy of marketing science*, 16(1), 74–94.

[82] Hair J. F., Ringle C. M., & Sarstedt M. (2013). Partial least squares structural equation modeling: Rigorous applications, better results and higher acceptance. *Long range planning*, 46(1–2), 1–12.

[83] Shah M. A. R., Husnain M., & Zubairshah A. (2018). Factors affecting brand switching behavior in telecommunication industry of Pakistan: A qualitative investigation. *American journal of industrial and business management*, 8(2), 359–372.

[84] Collins J. (2001b). Level 5 Leadership: The Triumph of Humility and Fierce Resolve. *Harvard Business Review*. 4(1), 67–76. 11189464

[85] Gorden W. I. (1988). Range of employee voice. *Employee Responsibilities and Rights Journal*, 1(4), 283–299.

[86] Yee L. S., Sandaran S. C., & Razak S. S. A. (2018). Employee Voice and the Communication of Dissent in an Organisational Setting in Malaysia: A Case Study. *LSP International Journal*, 5(1), 23–47.

[87] Gruman J. A., & Saks A. M. (2020). Employee and collective voice engagement: Being psychologically present when speaking up at work. In *Handbook of Research on Employee Voice*. Edward Elgar Publishing.

[88] Van D., Cummings L., & Parks J. (1995). Extra-role behaviors: In pursuit of construct and definitional clarity (a bridge over muddied waters). *Research in organizational behavior*, 17, 215–285.

[89] Graham J. W. (1991). An essay on organizational citizenship behavior. *Employee Responsibilities and Rights Journal*, 4(4), 249–270.

[90] Collins J. (2006). Level 5 leadership: The triumph of humility and fierce resolve. *Managing Innovation and Change*, 234. 11189464

